# Delayed versus on arrival modified live viral vaccination in stocker cattle on bovine respiratory disease

**DOI:** 10.18849/ve.v7i1.503

**Published:** 2022-03-04

**Authors:** Ashlee Ambs+, Heather Moberly+, Sarah Capik+

**Keywords:** BRD, CATTLE, MODIFIED LIVE VACCINE, MORBIDITY, STOCKER, VACCINE TIMING

## Abstract

PICO question

In auction market calves at high risk of developing bovine respiratory disease (BRD), does delayed (14–30 days) vaccination with a modified live vaccine (MLV) for viral respiratory pathogens versus administration of MLV on arrival (within 24 hours of arrival) to the stocker operation, result in less calves with BRD morbidity diagnosed based on visual signs and rectal temperature >40°C, or less calves with BRD mortality?

Clinical bottom line

Category of research question

Treatment

The number and type of study designs reviewed

Four papers were critically reviewed. All were randomised complete block designs

Strength of evidence

Moderate

Outcomes reported

In stocker calves, delaying administration of a MLV for respiratory viruses may result in numerically lower initial BRD morbidity rates, while giving at arrival may result in numerically lower BRD retreatments. One study shows statistically lower cases of BRD morbidity after the third antimicrobial treatment in cattle vaccinated on arrival with both a clostridial and MLV for respiratory viruses compared to cattle vaccinated on arrival with clostridial vaccine and delayed MLV for respiratory viruses. No conclusion about mortality can be drawn due to inconsistent numerical conclusions between studies

Conclusion

Due to conflicting evidence and a general lack of statistically significant differences in morbidity and mortality outcomes, a definite answer regarding the impact of delayed MLV respiratory vaccination in stocker calves cannot be made

## How to apply this evidence in practice

The application of evidence into practice should take into account multiple factors, not limited to: individual clinical expertise, patient’s circumstances and owners’ values, country, location or clinic where you work, the individual case in front of you, the availability of therapies and resources.

Knowledge Summaries are a resource to help reinforce or inform decision making. They do not override the responsibility or judgement of the practitioner to do what is best for the animal in their care.

## Clinical scenario

Stocker cattle are young and lightweight calves (often somewhere in the range of 204–250 kg) purchased to utilise pasture for frame-growth before moving to a feedlot for the finishing phase of their production life. Since this is often also a situation where recently weaned calves are commingled, bovine respiratory disease (BRD) is a concern. Modified live vaccines (MLV) containing viral respiratory pathogens are commonly administered to calves on arrival to stocker operations as a way to manage BRD risk. Recently, vaccine protocols have been reexamined in the interest of reducing BRD morbidity and several research projects have explored changing the timing of vaccine administration. In particular, delaying MLV administration for viral respiratory pathogens may allow an adjustment period for calves before challenging their immune system with a live vaccine. Stocker operations and veterinarians should review the research evidence and consider the financial and production impact of any changes in MLV timing on their operations.

## The evidence

There is no statistical evidence that delayed MLV administration for viral respiratory pathogens reduces BRD morbidity or mortality in stocker calves. While there is some evidence of numerical reductions in BRD morbidity, there is disagreement in the numerical differences in retreatment and mortality rates between arrival and delayed vaccinated calves between studies.

## Summary of the evidence

**Poe et al. (2013):** 

Population:	High-risk beef steer and bull calves at 202 ± 4.1 kg.
Sample size:	n = 393, (15–23 calves / pen, seven pens / treatment).
Intervention details:	Treatments: On arrival respiratory vaccination (day 0) with growth-promoting implant. On arrival respiratory vaccination (day 0) without growth-promoting implant. Delayed respiratory vaccination (day 14) with growth-promoting implant. Delayed respiratory vaccination (day 14) without growth-promoting implant. Vaccine: Pentavalent modified live vaccine (MLV) containing infectious bovine rhinotracheitis virus (IBRV), bovine viral diarrhoea virus (BVDV) type I and II, bovine respiratory syncytial virus (BRSV), parainfluenza-3 virus (Pl_3_); (Bovi-Shield GOLD® 5, subcutaneously; Pfizer Animal Health). Metaphylaxis: None, but calves could receive treatment with florfenicol if their rectal temperature was ≥40°C during processing regardless of clinical signs.
Study design:	Randomised complete block design with a 2x2 treatment factorial.
Outcome studied:	Morbidity measured as: First BRD treatment % (cattle exhibiting ≥2 clinical signs of BRD (ocular or nasal discharge, depressed appearance, gaunt or lacking normal fill, coughing, laboured breathing, or lack of appetite were pulled and rectal temperature taken, and antimicrobial treatment was administered if rectal temperature was ≥40°C) – subjective assessment; unblinded personnel. Second, third, and fourth BRD treatment % (cattle with symptoms of BRD and a rectal temperature of ≥40°C at 72 hours after the previous treatment) – subjective assessment; unblinded personnel.
Main Findings (relevant to PICO question)	BRD-related morbidity did not differ statistically due to vaccine timing.
Limitations:	No blinding of personnel. Pens had varying numbers of calves between blocks. The study reports that if any steers were identified as BRD cases >21 days following their previous treatment, they were restarted on the BRD treatment protocol as if they had never been diagnosed previously and considered a new incident case of BRD. However, it is unclear how new incidence of BRD after >21 days without clinical signs was managed statistically and if / where those animals are represented in the results. Study did not evaluate mortality. It is unclear whether calves were processed immediately upon arrival or after an overnight rest following arrival at the facility. There appear to be two acceptable case definitions that could have qualified calves for first treatment for BRD. One being having a rectal temperature ≥40°C at processing, regardless of the presence of other symptoms, and the other having ≥2 clinical signs of BRD plus meeting temperature criteria as outlined above after day 0 processing. This makes interpreting BRD incidence and comparison between studies potentially complex. This study did not describe any efforts to reduce potential cross-contamination between vaccinated and unvaccinated calves during treatment application, housing, subsequent sampling, and evaluation for and treatment of morbid animals.

**Richeson et al. (2008):** 

Population:	High-risk beef steer and bull calves at 197 ± 2.4 kg.
Sample size:	n = 528; (10–19 animals/pen, 18 pens/treatment).
Intervention details:	Treatments: Initial vaccination on arrival processing (day 0) with a booster at day 14. Delayed initial vaccination (day 14) with a booster at day 28. Vaccine: Pentavalent modified live vaccine (MLV) for respiratory pathogens containing infectious bovine rhinotracheitis virus (IBRV), bovine viral diarrhoea virus (BVDV) types I and II, bovine respiratory syncytial virus (BRSV), and parainfluenza-3 virus (Pl_3_) in combination; (EXPRESS 5, subcutaneously; Boehringer-Ingelheim Vetmedica Inc). Metaphylaxis: Calves received tilmicosin for metaphylaxis only if they had a rectal temperature of ≥40°C at arrival processing and were not included in subsequent morbidity outcomes.
Study design:	Randomised complete block design.
Outcome studied:	Bovine respiratory disease (BRD) morbidity in the first 42 days on feed (cattle with visual symptoms of respiratory illness -depression, lethargy, rapid breathing, nasal or ocular discharge, slowness in going to feed bunk, and a gaunt or emaciated appearance- and with a rectal temperature ≥40°C – subjective assessment by blinded personnel. Second and third morbidity (cattle were re-evaluated 72 hours after first or second treatment and were eligible for retreatment if they had a rectal temperature ≥40°C at that time) – objective assessment by blinded personnel. Percentage of death loss over 56 day study.
Main Findings (relevant to PICO question)	No statistically significant differences were observed. Calves that received the MLV on arrival processing had higher initial BRD morbidity (71.65% vs 63.5%; standard error (SE) 7.61; p-value: 0.12) but required less second treatments than cattle that received the MLV on day 14 (25.1% vs 30.8%; SE 9.80; p-value: 0.17), and also experienced increased death loss numerically (2.3% vs 0.8%; SE 0.75; p-value: 0.16).
Limitations:	Animals per pen varies almost two-fold. Unclear whether reported death loss is BRD-specific or overall mortality. Case definition is vague as there is no description of how many clinical signs were required for first treatment and it is unclear whether any clinical signs (other than a rectal temperature above their cut-off) were required in order to be eligible for second or third treatment. Numerical differences are difficult to interpret in a meaningful manner and are not necessarily broadly applicable or repeatable. Confidence intervals for estimates were not provided. Cattle were received on day -1 and ear tags were placed and weights were obtained prior to arrival processing on day 0. This study did not describe any efforts to reduce potential cross-contamination between vaccinated and unvaccinated calves during treatment application, housing, subsequent sampling, and evaluation for and treatment of morbid animals.

**Richeson et al. (2009):** 

Population:	High-risk beef steer and bull calves at 239 ± 1.2 kg.
Sample size:	n = 263 (10–12 calves/pen, six pens/treatment).
Intervention details:	Vaccines: CLOS: Clostridium chauvoei, Clostridium septicum, Clostridium novyi, and Clostridium sordellii and Clostridium perfringens types C & D bacterin- toxoid in a special oil adjuvant; Alpha-7, subcutaneously; Boeringer Ingleheim Vetmedica. RESP: Pentavalent modified live vaccine (MLV) containing infectious bovine rhinotracheitis virus (IBRV), bovine viral diarrhoea virus (BVDV) type 1a and type 2a, parainfluenza-3 virus (Pl_3_), and bovine respiratory syncytial virus (BRSV); (EXPRESS 5, subcutaneously; Boeringer Ingelheim Vetmedica). Treatments: ACAR: Arrival CLOS (day 0) and arrival (day 0) RESP. ACDR: Arrival CLOS (day 0) and delayed RESP (day 14). DCAR: Delayed CLOS (day 14) and arrival RESP (day 0). DCDR: Delayed CLOS (day 14) and delayed (day 14) RESP. A RESP vaccine booster was given 14 days after initial dose in all treatment groups. Metaphylaxis: All calves received tilmicosin metaphylaxis on arrival with a 48 hour post-treatment interval where they were not eligible for bovine respiratory disease (BRD) treatment.
Study design:	Randomised complete block design with a 2x2 treatment factorial.
Outcome studied:	BRD morbidity rate (cattle with two or more visual signs of BRD with rectal temperature ≥40°C) – subjective assessment, unblinded personnel. Second BRD treatment (cattle with rectal temperature ≥40°C at 48 hours after initial treatment – objective assessment, unblinded personnel. Third BRD treatment (cattle with rectal temperature ≥40°C at 72 hours after second treatment – objective assessment, unblinded personnel. Percentage of chronic animals (displayed BRD symptoms after third antibiotic treatment) – subjective assessment; unblinded personnel. Percentage of dead (not BRD-specific) in 56-day study.
Main Findings (relevant to PICO question)	No statistical differences between delayed versus arrival vaccine administration in percentage of dead calves (RESP p-value: 0.7). BRD morbidity rates were not statistically different but were numerically less in calves with delayed administration of the MLV (65.1%) versus those vaccinated on arrival (73.4%; no standard error of the mean (SEM) reported for RESP contrast; RESP p-value: 0.23). Percentage of chronic animals (cattle that displayed signs of BRD after the third antibiotic treatment) was greatest statistically in cattle administered clostridial vaccine on arrival and delayed respiratory vaccine (ACDR: 11.4%) compared to those receiving clostridial vaccine and respiratory vaccine on arrival (ACAR: 1.5%; p=0.04), and numerically higher than those receiving a delayed clostridial vaccine with either arrival (DCAR: 6.3%; P = 0.26) or delayed respiratory vaccine (DCDR: 3.2%; P = 0.08). The SEM was 4.7 with the interaction between CLOS and RESP having a p-value of 0.05. The percentage of animals treated a second time for BRD out of the total population did not differ significantly between groups (ACAR: 38.5%, ACDR: 39.5%, DCAR: 36.1%, DCDR: 25.5%; SEM: 8.7; RESP p-value: 0.43; interaction p-value: 0.34). The percentage of animals treated a third time for BRD out of the total population did not differ significantly between groups (ACAR: 20.5%, ACDR: 22.4%, DCAR: 19.8%, DCDR: 10.8%; SEM: 9.4; RESP p-value: 0.61; interaction p-value: 0.43).
Limitations:	No blinding of evaluators for subjective outcomes. Unclear whether reported death loss is BRD-specific or overall mortality. Numerical differences are difficult to interpret in a meaningful manner and are not necessarily broadly applicable or repeatable. Overall means in delayed vs arrival RESP were not provided for all outcomes. There were no SEMs available for main effects. Confidence intervals for estimates were not provided. Cattle appear to have been processed the same day they arrived at the facility. This study indicated that fenceline contact between vaccinated and non-vaccinated calves was possible. It did not describe efforts to reduce potential cross-contamination between vaccinated and unvaccinated calves during treatment application, subsequent sampling, and evaluation for and treatment of morbid animals.

**Richeson et al. (2015):** 

Population:	High-risk beef steer and bull calves at 211 ± 2.6kg in Fall, 213 ± 5.4kg in Spring.
Sample size:	n = 184 Fall, 186 Spring (two blocks / season, two pens / treatment / block).
Intervention details:	Treatments: AMLV: Initial modified live respiratory pathogen vaccine (MLV) on day 0. DMLV: Initial MLV vaccine on day 14. NMLV: No vaccination with MLV vaccine until the end of the 42-day receiving period. Both AMLV and DMLV calves were revaccinated 14 days after initial vaccination. Vaccine: Pentavalent modified live vaccine (MLV) containing infectious bovine rhinotracheitis virus (IBRV), bovine viral diarrhoea virus (BVDV) types I and II, parainfluenza-3 virus (Pl_3_), and bovine respiratory syncytial virus (BRSV); (Bovi-Shield Gold 5, unspecified route; Zoetis). Metaphylaxis: All calves received tilmicosin metaphylaxis on arrival with a 24 hour post-metaphylaxis interval observed during which they were not eligible to be treated for bovine respiratory disease (BRD).
Study design:	Randomised complete block design.
Outcome studied:	Percentage of cattle diagnosed with BRD at least once (cattle observed with ≥2 visual signs of BRD (depression, lethargy, rapid breathing, nasal or ocular discharge, and lack of appetite) and a rectal temperature ≥40°C were considered morbid and were treated) – subjective assessment, blinded personnel. Relapses (percentage of cattle treated for BRD that required ≥ 1 retreatment; counted once for statistical analysis) – subjective assessment, blinded personnel: Cattle were eligible for retreatment 72 hours after previous treatment if they again had ≥2 visual signs of BRD and a rectal temperature of ≥40°C. Each animal was eligible for up to five total treatments for BRD. Percentage of mortality associated with clinical BRD in 42-day study.
Main Findings (relevant to PICO question)	Morbidity associated with BRD was not significantly affected by MLV timing (AMLV: 35.4% vs DMLV: 34.9%; standard error (SE) of the least square means (LSM): 11.2; p-value = 0.94), however a lower percentage of delayed vaccination calves was considered morbid and relapsed, than calves who were vaccinated on arrival (DMLV: 23.3% vs AMLV: 30.1%; SE of LSM: 9.1; p-value = 0.51). Though not statistically evaluated, the percentage of calves treated three times for BRD was lower in arrival vaccination calves (0.5%) versus delayed (3.6%). Mortality percentage was higher numerically in calves receiving delayed vaccination than those vaccinated on arrival, but not statistically significant (DMLV: 1.6% vs AMLV: 0.9%; SE of LSM: 1.1; p-value = 0.67).
Limitations:	Blocks did not contain the same number of calves (Block 1 = 93; Block 2 = 91; Block 3 = 71; Block 4 = 118) and the number of calves in each pen within each block was not specified. Authors did not indicate the route of vaccine administration. Numerical differences are difficult to interpret in a meaningful manner and are not necessarily broadly applicable or repeatable. Confidence intervals for estimates were not provided. Cattle appear to have been processed the same day they arrived at the facility. This study did not describe efforts to reduce potential cross-contamination between vaccinated and unvaccinated calves during treatment application, housing, subsequent sampling, and evaluation for and treatment of morbid animals.

## Appraisal, application and reflection

Overall, we identified no statistical evidence that delayed modified live vaccination (MLV) for viral respiratory pathogens lowered bovine respiratory disease (BRD) morbidity or mortality rates. One study (Richeson et al., 2009) indicated statistically lower cases of BRD morbidity in chronic animals (displaying clinical signs of BRD after the third antimicrobial treatment) vaccinated on arrival with both a clostridial vaccine and MLV for respiratory viruses compared to cattle vaccinated on arrival with clostridial vaccine and delayed MLV for respiratory viruses. However, there were several interesting numerical differences in initial BRD morbidity favouring delayed vaccination in several of the studies that suggest perhaps additional, well-powered studies on this question are warranted. Mortality and retreatment numerical differences were inconsistent between studies. Additionally, there were numerous other differences between studies including BRD case definition, vaccines used, different treatment regimens employed, and other management differences that made comparing results between studies difficult.  Although we did not specifically limit our search geographically, all four studies that met our criteria and are discussed in this Knowledge Summary were performed in the United States.

Although, numerical differences cannot be used to support study conclusions they are important to report for discussion. Two studies showed numerical differences in retreatment and relapses (Richeson et al., 2009; and Richeson et al., 2015). In one study (Richeson et al., 2015) with blinded observers, all cattle that relapsed and required retreatment were counted once regardless of the number of times this occurred, while in the other (Richeson et al., 2009) which did not have blinded observers, retreatment rates were measured as a percentage treated with second or third antibiotic treatments and each retreatment was analysed individually. Including all the relapses in one analysis, as in Richeson et al. (2015), does not allow evaluation of multiple retreatments and limits our ability to compare this study to others that specifically separate evaluations of subsequent treatments. Given the potential impact that delayed vaccination could have on the resulting immune responses of the cattle, it would be beneficial to evaluate second and third treatment rates separately.

Limitations related to study design, such as lack of blinding or small sample sizes, increase the chances for biased results and the results, especially numerical differences, must be interpreted with caution. Bovine respiratory disease morbidity is inherently a subjective assessment of health, because identifying a potentially ill animal is largely accomplished via visual signs and sometimes followed by an objective measurement of temperature. Blinding of individuals that are evaluating subjective outcomes like BRD morbidity helps prevent unintentional biases that could impact those subjective outcomes in unpredictable ways. Two of the four papers evaluated (Poe et al., 2013; and Richeson et al.,2009), indicated that evaluators were not blinded when evaluating subjective outcomes. Three of the four papers evaluated had varying, or uneven, numbers of animals in pens or between blocks among treatments (Poe et al., 2013; Richeson et al., 2008; and Richeson et al., 2015). It remains uncertain whether the density of a pen influences the risk of BRD for the animals in it, potentially due to a decrease in available space and resources or due to stress from the commingling experience. Additionally, three of the studies (Poe et al. 2013; Richeson et al., 2009; and Richeson et al., 2015) had smaller sample sizes than the other (Richeson et al., 2008) which may have impacted their ability to detect smaller differences in morbidity.

Even though each study provided evidence for a very specific question, there are multiple differences between the four studies that must be considered when interpreting the results. A notable difference is that two studies (Richeson et al., 2008; and Richeson et al., 2009) evaluated one MLV for respiratory viruses (EXPRESS 5) while the other two studies (Poe et al., 2013; and Richeson et al., 2015) evaluated another MLV for respiratory viruses (BoviShield Gold® 5). These are only two of multiple MLV for respiratory viruses available on the market in the US and there are differences in the adjuvant used, different pathogen loads, different virus types and strains included, routes of administration, etc., between manufacturers and vaccines that could impact the results. Therefore, although the studies provide evidence for our question related to delayed versus on-arrival MLV use, the evidence each provides is not necessarily externally valid when considering all MLV for respiratory viruses. Another area of variability between studies is the difference in the definition of ‘arrival’. Two studies appear to have processed calves on the day they arrived (Richeson et al., 2009; and Richeson et al., 2015); one indicates they were processed the day after they arrived (Richeson et al., 2008), and one seems to indicate calves were processed after resting ‘overnight’ on grass and hay (Poe et al., 2013). Further potential variability exists regarding the amount of potential cross-contamination possible via direct or fomite contact between calves that were and were not vaccinated in each study during the period when they may have shed vaccine virus; mitigation strategies were not well-described. Treatment and prophylaxis regimens are an additional area of variability in these protocols and throughout different feeding operations; it is possible that the impact of delayed vaccination could vary between operations due to differences in these other important health management factors.

The two studies that used the EXPRESS 5 MLV (Richeson et al., 2008; and Richeson et al., 2009) gave prophylactic tilmicosin and treated for internal and external parasites on arrival to all cattle, and boostered the MLV 14 days after they were first administered. However, one study (Richeson et al., 2009) was also evaluating timing of clostridial vaccine while the other (Richeson et al., 2008) gave all calves a clostridial vaccine on arrival. For prophylaxis, one (Richeson et al., 2008) only administered tilmicosin if arrival rectal temperature was ≥40°C and those cattle were excluded from further morbidity assessment, while the other (Richeson et al., 2009) administered it to all cattle on arrival and all were able to be evaluated for morbidity the next day. There was not a statistical difference in the number of cattle in each treatment that were excluded in Richeson et al. (2008), which should have prevented a differential bias in morbidity outcomes between vaccine treatments. However, those cattle appear to have remained with their pen mates who did not receive prophylaxis, which may have differentially influenced the morbidity during the study in general and especially when compared to studies that provide metaphylaxis to all calves equally. These differences in arrival management between the two studies makes them difficult to compare. Additionally, in both studies, cattle were able to be evaluated for retreatment due to BRD morbidity after 72 hours, when they could be administered a different antibiotic. Each allowed up to three retreatments. However, each study used different antibiotics as their first, second, and third treatments (Richeson et al. [2008] used tilmicosin, enrofloxacin, and florfenicol, respectively; Richeson et al. [2009] used florfenicol, ceftiofur, and danofloxacin, respectively), which also makes comparison of retreatment rates between studies difficult.

The two studies that evaluated BoviShield Gold® 5 MLV (Poe et al. 2013; and Richeson et al., 2015) administered a clostridial vaccine and treated for internal and external parasites on arrival to all animals but differed in other parts of their protocols. For prophylaxis, Richeson et al. (2015) administered tilmicosin to all cattle on arrival and cattle were able to be evaluated for BRD morbidity after 24 hours. In Poe et al. (2013) cattle with a rectal temperature that was ≥40°C at processing received their first treatment of florfenicol at processing regardless of clinical signs. They were presumably then able to be reevaluated after 72 hours when they could receive the second antibiotic treatment in their protocol, though this was not explicitly described. However, cattle who were not treated at processing seemed to be eligible to be evaluated the next day to determine if initial BRD treatment should be administered. Additionally, it does not appear that treatment during processing was a criteria for randomising nor is it clear whether delayed or arrival vaccinated calves had similar incidences of calves treated at processing. The differential treatment of calves in this study complicates assessment of BRD incidence. In both papers, morbid cattle could be evaluated for retreatment due to BRD morbidity after 72 hours, when they could be administered a different antibiotic (Richeson et al. [2015] used florfenicol, enrofloxacin, tilmicosin, ceftiofur, then tulathromycin; Poe et al. [2013] used florfenicol, tilmicosin, enrofloxacin, then tulathromycin).However, one study (Poe et al., 2013) described that cattle going >21 days following treatment before exhibiting additional BRD symptoms, were considered a new case of BRD and their treatment regimen began again at florfenicol. Selection of an antibiotic protocol is often a producer or veterinarian preference based on known efficacy, availability, pharmacology, etc. Due to the different spectrums and modes of action associated with these drugs their use as metaphylaxis or treatment may impact BRD morbidity outcomes (O’Connor et al., 2019).

Case definition and criteria that make a calf eligible for BRD treatment or retreatment was also a notable difference between studies that complicated comparisons. Poe et al. (2013) actually used two different case definitions, one during processing where calves could receive treatment if they had a rectal temperature ≥40°C regardless of clinical signs, and then after day 0 when they had to have ≥2 clinical signs plus a rectal temperature of ≥40°C. Richeson et al. (2008) required an unspecified number of visual signs plus rectal temperature of ≥40°C for initial treatment while Richeson et al. (2009; and 2015) required ≥2 clinical signs plus a rectal temperature of ≥40°C to be eligible for initial treatment. Retreatment criteria also varied between studies with some requiring some form of clinical signs plus a temperature threshold to be met (Poe et al., 2013; and Richeson et al., 2015) while others evaluated only the rectal temperature of calves at predetermined intervals after initial or subsequent treatment (Richeson et al., 2008; and 2009). These different criteria, while externally valid, highlight one of the reasons why repeatability of BRD research is so difficult to realise and how variable identification and management of BRD cases can be within the literature.

All four studies evaluated male calves that were either procured already castrated or castrated on arrival. Although they all accounted for castration status in their analyses, three of the four studies (Poe et al., 2013; Richeson et al., 2008; and Richeson et al., 2015) used a California banding technique for castration while the other study (Richeson et al., 2009) used surgical castration. Cattle react differently to alternate castration methods and the variable inflammatory response plus the impact castration can have on clinical signs such as depression, decreased appetite, etc., could alter the impact of delayed vaccination in a commercial setting (Roberts et al., 2018). The lack of representation of heifers in the study populations also limits our ability to extrapolate these results to high risk heifer calves in stocker operations.

Mortality was evaluated in three studies (Richeson et al., 2008; Richeson et al., 2009; and Richeson et al., 2015) but none found a statistical difference between delayed versus on arrival vaccine administration treatment groups. In Richeson et al. (2009) there were inconsistent numerical differences due to the added variable of clostridial vaccine timing. The other two studies had opposing numerical differences, where one (Richeson et al., 2008) showed a higher percentage of death loss over the 56 day study in calves vaccinated on arrival and the other (Richeson et al., 2015) had a higher percentage of mortality associated with clinical BRD in delayed vaccination calves during the 42 day study. The latter study revealed an association with BRD and death loss while the former did not describe a cause for death. Therefore, it cannot be concluded that all deaths were related to BRD making comparison of the two studies more complicated. Since these studies disagreed numerically, it is important to recognise these differences as well as those previously mentioned, such as the use of prophylaxis in Richeson et al. (2015) which was not used in Richeson et al. (2008). Given the small percentages in death loss among the treatments in these studies, which ranged from 0.8–2.3%, it is also possible that they were underpowered to truly evaluate mortality.

Even with a similar goal for these studies and some with the same vaccines administered, there are many aspects of a protocol that can introduce variability and make it difficult to interpret outcomes. Producers and veterinarians should use the information provided in this summary to make vaccine protocol decisions considering the limitations listed above. Since the evidence differed among studies and no statistical difference between arrival versus delayed vaccine administration was identified, no answer can be given to the clinical question.

## Methodology Section

**Search Strategy:** 

Databases searched and dates covered:	CAB Abstracts on OVID platform: 1910–2021 Week 42 Search run with no limits or filters Limit results to English and 2000–current PubMed on NCBI Website: 1946–27 October 2021 Search run with no limits or filters Limit results to English and 2000–current
Search strategy:	CAB Abstracts: ((exp cattle/ or (calf or calves or steer or steers or heifer or heifers or bull or bulls or bovine or bovines or cattle or youngstock or young-stock or (young adj2 stock)).mp.) and (exp vaccines/ or vaccin*.mp. or exp immunization/ or immuni*.mp.) and (delay or delays or delayed or arrive or arrives or arrived or arrival or postarrival or "post arrival" or post-arrival).mp. and (pneumon*.mp. or (respiratory adj1 disease*).ti,ab. or "respiratory diseases".sh. or (respiratory adj2 disease*).ti,ab. or ((shipping or undifferentiated) adj1 fever).ti,ab. or (BRD or BRDC).ti,ab. or (bovine adj1 respiratory adj1 disease*).ti,ab. or (bovine adj1 respiratory adj1 disease* adj1 complex).ti,ab. or (summer adj1 pneumon*).ti,ab. or (enzootic adj1 pneumon*).ti,ab. or pleuropneumon*.mp. or bronchopneumon*.mp. or (respiratory adj1 tract adj1 disease*).mp.)) PubMed: (("pneumonia"[MeSH Terms] OR "pneumonia"[Title/Abstract] OR "pneumoniae"[Title/Abstract] OR "pneumonias"[Title/Abstract] OR "respiratory diseases"[All Fields] OR "respiratory disease"[Title/Abstract] OR "shipping fever"[Title/Abstract] OR "undifferentiated fever"[Title/Abstract] OR "BRD"[Title/Abstract] OR "BRDC"[Title/Abstract] OR "bovine respiratory disease"[Title/Abstract] OR "bovine respiratory disease complex"[All Fields] OR "summer pneumonia"[Title/Abstract] OR "enzootic pneumonia"[Title/Abstract] OR ("pleuropneumonia"[MeSH Terms] OR "pleuropneumonia"[Title/Abstract] OR "pleuropneumonias"[Title/Abstract] OR "pleuropneumoniae"[Title/Abstract]) OR ("bronchopneumonia"[MeSH Terms] OR "bronchopneumonia"[Title/Abstract] OR "bronchopneumonias"[Title/Abstract] OR "bronchopneumoniae"[Title/Abstract]) OR "respiratory tract disease"[Title/Abstract] OR "respiratory tract diseases"[All Fields]) AND ("calf"[Title/Abstract] OR "calves"[Title/Abstract] OR "steer"[Title/Abstract] OR "steers"[Title/Abstract] OR "heifer"[Title/Abstract] OR "heifers"[Title/Abstract] OR "bull"[Title/Abstract] OR "bulls"[Title/Abstract] OR "bovine"[Title/Abstract] OR "bovines"[Title/Abstract] OR "cattle"[Title/Abstract] OR "cattle"[MeSH Terms] OR "youngstock"[Title/Abstract] OR "young stock"[Title/Abstract] OR "young-stock"[Title/Abstract]) AND ("immunization"[MeSH Terms] OR "immunization"[Title/Abstract] OR "immunisation"[Title/Abstract] OR "immunizations"[Title/Abstract] OR "immunisations"[Title/Abstract] OR "immunize"[Title/Abstract] OR "immunise"[Title/Abstract] OR "immunized"[Title/Abstract] OR "immunised"[Title/Abstract] OR "vaccination"[MeSH Terms] OR "vaccine"[Title/Abstract] OR "vaccines"[Title/Abstract] OR "vaccination"[Title/Abstract] OR "vaccinating"[Title/Abstract] OR "vaccinated"[Title/Abstract]) AND ("delay"[Title/Abstract] OR "delayed"[Title/Abstract] OR "delays"[Title/Abstract] OR "post-arrival"[Title/Abstract] OR "post-arrival"[Title/Abstract] OR "postarrival"[Title/Abstract] OR "arrive"[Title/Abstract] OR "arrival"[Title/Abstract] OR "arrives"[Title/Abstract] OR "arrived"[Title/Abstract])
Dates searches performed:	27 Oct 2021

**Exclusion / Inclusion Criteria:** 

Exclusion:	Publication date prior to 2000, is a systematic review and / or meta-analysis, is conference proceedings, does not evaluate the bovine species, does not compare arrival versus delayed vaccine administration groups, does not evaluate the same vaccine given on arrival versus delayed administration, the delayed time was less than 14 days post-arrival, does not evaluate BRD morbidity, or calves not evaluated in a stocker operation.
Inclusion:	BRD morbidity assessment, respiratory vaccine used when evaluating vaccine timing, and comparison of effects of delayed modified live vaccine administration and administration of vaccine on arrival to the stocker operation.

**Search Outcome:** 

Database	Number of results	Excluded – Not a research trial	Excluded – Not an MLV respiratory vaccine study in calves	Excluded – Does not compare arrival versus delayed (14–30 days) administration of the same MLV vaccine	Excluded – Calves not evaluated or administered treatment at stocker operation	Total relevant papers
CAB Abstracts	94	17	35	35	3	4
PubMed	50	7	16	24	1	2
Total relevant papers when duplicates removed						4

## Conflict of Interest

- Ambs was advised and led by Dr. John Richeson (one of the authors or co-authors of all papers that are evaluated within this summary) to obtain her Master’s degree at West Texas A&M University from January 2016 to May 2017.
- Capik collaborates regularly with Dr. John Richeson (one of the authors or co-authors of all papers that are evaluated within this summary) and has collaborated with two of the co-authors on Richeson et al. 2009 (Kegley and Powell) and on co-author on Poe et al. 2013 (Kegley).
- Moberly serves on the CABI Publishing North American Library Advisory Board and the VetStream Academic Advisory Board.

## Disclaimer

Knowledge Summaries are a peer-reviewed article type which aims to answer a clinical question based on the best available current evidence. It does not override the responsibility of the practitioner. Informed decisions should be made by considering such factors as individual clinical expertise and judgement along with patient’s circumstances and owners’ values. Knowledge Summaries are a resource to help inform and any opinions expressed within the Knowledge Summaries are the author’s own and do not necessarily reflect the view of the RCVS Knowledge. Authors are responsible for the accuracy of the content. While the Editor and Publisher believe that all content herein are in accord with current recommendations and practice at the time of publication, they accept no legal responsibility for any errors or omissions, and make no warranty, express or implied, with respect to material contained within. For further information please refer to our Terms of Use.
